# Integrating bioinformatic resources to predict transcription factors interacting with *cis*-sequences conserved in co-regulated genes

**DOI:** 10.1186/1471-2164-15-317

**Published:** 2014-04-28

**Authors:** Christian Dubos, Zsolt Kelemen, Alvaro Sebastian, Lorenz Bülow, Gunnar Huep, Wenjia Xu, Damaris Grain, Fabien Salsac, Cecile Brousse, Loïc Lepiniec, Bernd Weisshaar, Bruno Contreras-Moreira, Reinhard Hehl

**Affiliations:** 1 INRA, Institut Jean-Pierre Bourgin, Saclay Plant Sciences, UMR1318, RD10, F-78026, Versailles, France; 2 AgroParisTech, Institut Jean-Pierre Bourgin, Saclay Plant Sciences, UMR1318, RD10, F-78026, Versailles, France; 3 Current address: Biochimie et Physiologie Moleculaire des Plantes, UMR 5004, INRA/CNRS/SupAgro-M/UM2, 34060 Montpellier Cedex 1, France; 4 Estación Experimental de Aula Dei/CSIC, Av. Montañana 1.005, 50059 Zaragoza, Spain; 5 Institut für Genetik, Technische Universität Braunschweig, Spielmannstr. 7, 38106 Braunschweig, Germany; 6 Department of Biology, Bielefeld University, Universitaetsstrasse 25, 33615 Bielefeld, Germany; 7 Fundación ARAID, calle María de Luna 11, 50018 Zaragoza, Spain

**Keywords:** Databases, *Arabidopsis thaliana*, *Physcomitrella patens*, Yeast one-hybrid, Microarray, Transcription factor, *cis*-element

## Abstract

**Background:**

Using motif detection programs it is fairly straightforward to identify conserved *cis*-sequences in promoters of co-regulated genes. In contrast, the identification of the transcription factors (TFs) interacting with these *cis*-sequences is much more elaborate. To facilitate this, we explore the possibility of using several bioinformatic and experimental approaches for TF identification. This starts with the selection of co-regulated gene sets and leads first to the prediction and then to the experimental validation of TFs interacting with *cis*-sequences conserved in the promoters of these co-regulated genes.

**Results:**

Using the PathoPlant database, 32 up-regulated gene groups were identified with microarray data for drought-responsive gene expression from *Arabidopsis thaliana*. Application of the binding site estimation suite of tools (BEST) discovered 179 conserved sequence motifs within the corresponding promoters. Using the STAMP web-server, 49 sequence motifs were classified into 7 motif families for which similarities with known *cis*-regulatory sequences were identified. All motifs were subjected to a footprintDB analysis to predict interacting DNA binding domains from plant TF families. Predictions were confirmed by using a yeast-one-hybrid approach to select interacting TFs belonging to the predicted TF families. TF-DNA interactions were further experimentally validated in yeast and with a *Physcomitrella patens* transient expression system, leading to the discovery of several novel TF-DNA interactions.

**Conclusions:**

The present work demonstrates the successful integration of several bioinformatic resources with experimental approaches to predict and validate TFs interacting with conserved sequence motifs in co-regulated genes.

## Background

In recent years large numbers of novel *cis*-regulatory sequences have been described that are conserved in stress-response genes, but the role of these elements and their binding transcription factors (TFs) remains unknown [1-3]. The identification of the binding TFs is therefore a major challenge for bioinformaticians and experimentalists. Using database-assisted analysis of *cis*-sequences it is possible to generate hypotheses on the nature of the binding TF [4], but the experimental validation of these predictions is often missing. The current work investigates how efficient a database-assisted approach leads to the prediction of the correct TF or TF-families that bind to conserved *cis*-sequences in co-regulated genes.

The identification of *cis*-regulatory sequences has been facilitated using bioinformatic and web-queryable resources [4-9]. One approach is the detection of *cis*-elements annotated in existing resources by performing a database-assisted analysis [10-12]. Another approach is the *de novo* discovery of conserved sequence patterns in sets of co-regulated genes without knowing if these sequences have been associated with a function before. This involves detection of over-represented sequences in the upstream region of co-regulated genes by using pattern mining programs such as MEME, AlignACE, CONSENSUS, Co-Bind, BioProspector, MITRA, or integrative frameworks such as BEST [13-19].

To discover conserved sequence motifs in promoters of up-regulated *A. thaliana* genes, BEST was applied in the work presented here. Co-regulated genes were identified using drought responsive microarray expression data annotated to the PathoPlant database [20,21]. The identified motifs were classified with STAMP [22] and compared to known *cis*-regulatory sequences annotated to the AthaMap, PLACE and AGRIS databases [23-28]. Furthermore, the newly developed footprintDB repository, built on top of 3D-footprint, was employed to predict interacting TFs [29,30]. To confirm bioinformatic predictions, 15 *cis*-sequences were used for the isolation of interacting TFs in a yeast one-hybrid screening. The specificity of each TF-DNA interaction was further validated in yeast. Furthermore, two TFs were used for the generation of synthetic factors that activate reporter gene expression under the control of synthetic promoters harbouring corresponding *cis*-sequences in a *P. patens* expression system.

## Results

### Identification of seven motif families in drought-responsive *A. thaliana* genes

**Figure 1:**
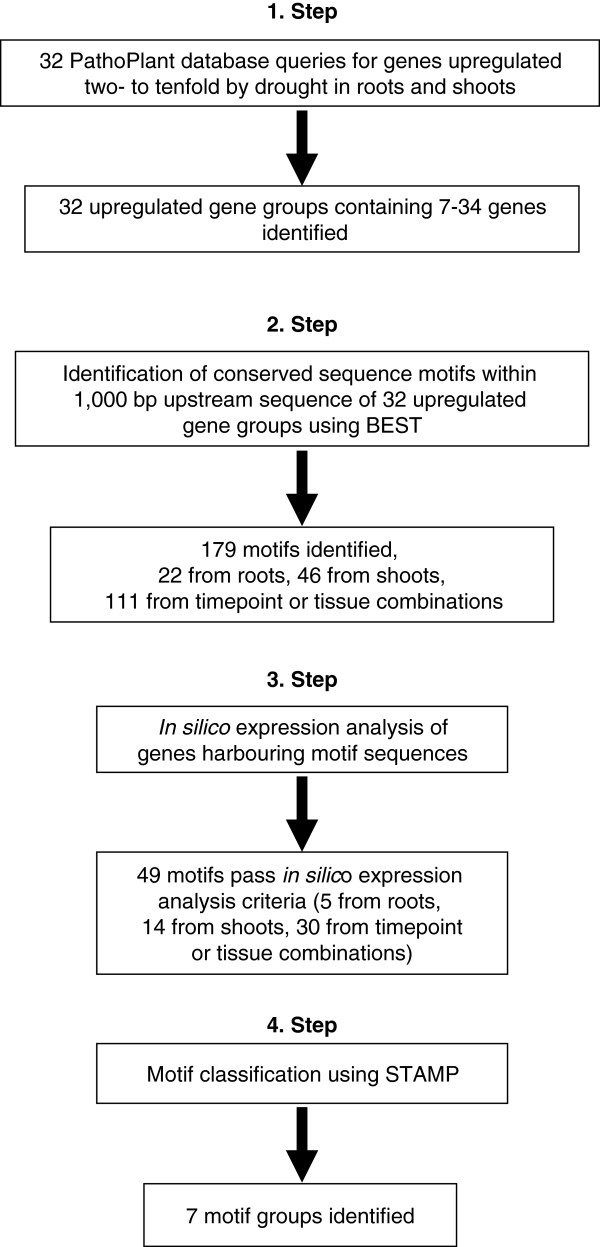
Workflow to identify sequence motifs conserved in drought responsive genes.

The goal of this work was the identification of transcription factors binding to *cis*-regulatory sequences conserved in promoters of drought-responsive genes. Thus, the first part aimed to identify sequence motifs harbouring such *cis*-sequences. Figure 1 gives an overview of the workflow. In a first step, microarray experiments annotated to the PathoPlant database were employed to identify gene sets two- to tenfold up-regulated by drought in roots and shoots. A total of 32 queries were performed. Additional file 1 lists the parameters for these queries and also the number of induced genes obtained in each of the 32 queries. The 32 queries yielded 32 up-regulated gene groups containing up to 34 co-regulated genes. In a second step, conserved sequence motifs within the upstream region of the 32 co-regulated gene groups were identified with the software package BEST [19]. For motif detection, 1,000 bp upstream of all genes in a co-induced gene group were screened with this software. This analysis resulted in 179 sequence motifs, 22 from roots, 46 from shoots, and 111 from combinations of timepoints or tissues. In a third step, all sequences from the 179 motifs were subjected to an *in silico* expression analysis [31]. With this tool, motifs specific for drought responsive genes were identified. A total of 49 motifs containing *cis-*sequences that pass this analysis remained, 5 from roots, 14 from shoots and 30 from combinations of timepoints or tissues. These sequences and the alignments generating the 49 sequence motifs are shown in Additional file 2. Single sequences that pass the *in silico* expression analysis criteria are shown in bold in this file.

**Figure 2:**
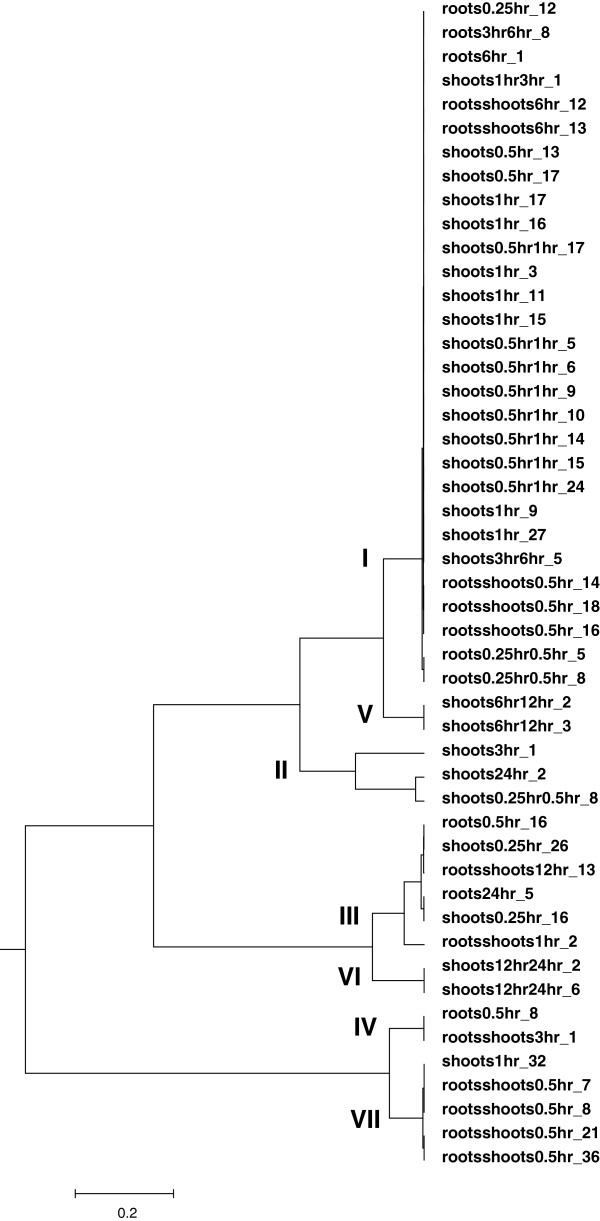
Relationship tree for 49 sequence motifs identified bioinformatically in co-regulated gene groups.

These 49 sequence motifs were further analysed using STAMP (Step 4, Figure 1). STAMP determines the relationship between the motifs and also calculates similarities to known *cis*-regulatory sequences [22]. To illustrate the similarities among the 49 sequence motifs, a tree was generated and illustrated by the program MEGA [32], which is shown in Figure 2. Based on the relationship between the motifs, 7 motif groups were designated. STAMP was also employed for the identification of motif similarities by comparing the 49 motifs with known *cis*-elements from plant databases AthaMap, AGRIS and PLACE [23-25]. Additional file 3 summarises these results. Several motifs from group I, that harbours 29 motifs, show significant similarities (low E-value) to a G-box motif and to abscisic acid-responsive elements (ABRE) known as bZIP binding sites. For example with the PLACE database eight motifs show similarities with the GBOXLERBCS sequence [33] and nine motifs show similarities to four different ABREs from PLACE (ABREATRD22, ABREAZMRAB28, ABRETAEM, ABREMOTIFIIIOSRAB16B). These ABREs are associated with abiotic stress response [34-37]. Twenty motifs cluster within the remaining groups (II to VII) and display less significant similarities (higher E-value) to a wide array of *cis*-elements, with many of them so far not being associated with abiotic stress response (Additional file 3).

### Bioinformatic prediction of TFs interacting with conserved *cis*-sequences

**Figure 3:**
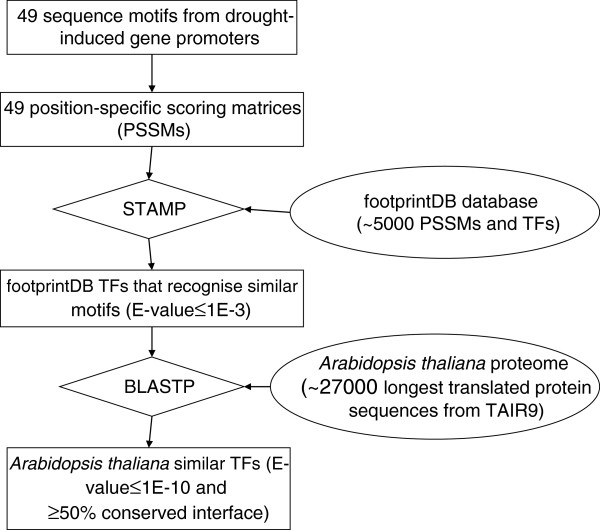
**Workflow to predict binding TFs for 49 *cis*-elements/sequence motifs.**

The collection of sequence motifs detected with BEST were further analysed with footprintDB, which currently contains over 5,000 unique TFs and their experimentally-associated DNA motifs [30]. The flowchart in Figure 3 illustrates this approach. This strategy retrieved *A. thaliana* candidates for all motif groups but group V. These predictions further confirm that 29 group I motifs display obvious similarities to G-box, ABRE-binding motifs recognised by TFs of the ABF family (with bZIP domains), and in general reproduce the STAMP analysis explained earlier. The remaining groups do not have a clear resemblance to any documented DNA binding specificity, and thus proteins from a variety of families were retrieved. In total, 865 unique *A. thaliana* protein sequences were retrieved for the 49 drought-related motifs (Additional file 4, sheet [A.th.homologues]).

### Isolation of TFs interacting with conserved *cis*-sequences

**Figure 4:**
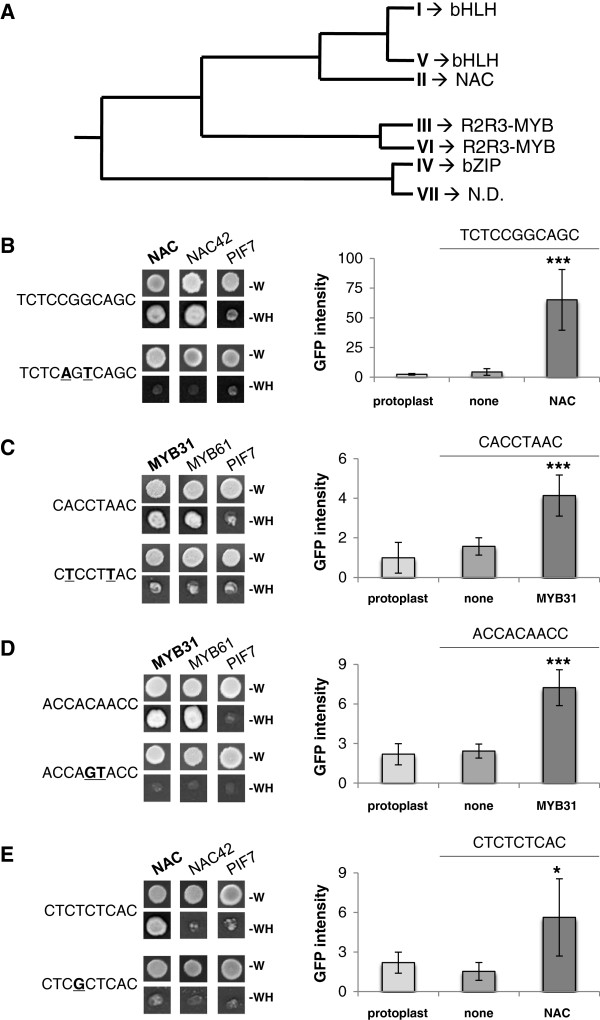
**Identification and functional characterization of TFs interacting with conserved *cis*-sequences. (A)** Simplified similarity tree (based on STAMP analysis) displaying the main associations observed between the different groups of conserved *cis*-sequences and the different class of TFs identified in yeast one-hybrid (Y1H) experiments. **(B-E)** Transcriptional activity and interaction of selected TFs with sequences 3 **(B)**, 7 **(C)**, 5 **(D)** and 9 **(E)** in Y1H experiments (left panels). Six repeats of the tested sequence, or their mutated versions (bold characters indicate the mutated nucleotides), fused to the *HIS3* auxotrophic gene were tested. Upper part, growth on control media deprived of W amino acid, allowing the selection of yeast that express the studied TFs (*i.e.* NAC, NAC42, PIF7, MYB61 and MYB31). Lower part, growth on selective media deprived of W and H amino acids. *Physcomitrella patens* protoplasts transient expression assays (right panels). Green fluorescent protein (GFP) intensity measured in *P. patens* protoplasts cotransfected with sequences 3, 7, 5 and 9 fused to the *35S* cauliflower mosaic virus minimal promoter and the *GFP* reporter gene with either, the NAC (sequences 3 and 9) or the MYB31 (sequences 7 and 5) DNA binding domain fused to the VP16 activation domain. Error bars ± SE. *t*-test significance: *, *P* < 0.05 and ***, *P* < 0.001.

Sequences from the identified DNA motifs, which are conserved in the promoter of genes simultaneously induced by drought were cloned (six repeats) into the yeast pHISi vector with the aim to carry out yeast one-hybrid (Y1H) screens, in order to identify TFs that are able to activate the transcription from these putative *cis*-regulatory sequences. Out of these 49 sequence motifs, 15 individual *cis*-sequences shown in Table 1 and representing all seven motifs groups, were used for Y1H. Additional file 5 shows the result of the Y1H screen. A total of 49 different TFs belonging to 12 different families were identified. Amongst the identified factors, 11 were already described as playing a role in various stress responses. Five were found to be associated with abiotic stresses (e.g. drought, salt), three with biotic stresses (e.g. pathogens), and three with both types of stress (Additional file 5). Moreover, this analysis led in most of the cases to the identification of specific associations between a motif group and a TF family. Table 2 and Figure 4A summarise these findings. Table 3 shows all selected TFs that were able to activate the screened sequence but unable to activate the mutated sequence in yeast (Figure 4B, C, D, and E, left panel). Sequence 2 from motif group I and sequence 10 from motif group V preferably select bHLH TFs (Table 2). Instead, both sequences from motif group II preferably select NAC factors. Interestingly, sequences from motif group III and VI, which are closely related (Figure 2), preferably select MYB TFs. For the other sequences, no preferred TF family members were selected (Table 2).

**Table 1:** ***Cis*-sequences selected for further experimental and bioinformatics analysis**

**Seq. Nr.**	**Selected *cis*-element**	**Motif group**	**Query**	**Gene**	**Position**
1	AACGTGGG	I	roots6h_1	At4g22610	−491
2	GCACGTGGAG	I	shoots1hr_9	At4g15210	−312
3	GCTGCCGGAGA	II	shoots24hr_2	At4g17470	−295
4	GCCACGTCAGC	II	shoots24hr_2	At5g05250	−526
5	GGTTGTGGT	III	roots24hr_5	At1g21100	−839
6	ACCAAACAT	III	shoots0.25hr_16	At5g42760	−26
7	CACCTAAC	III	roots0.5hr_16	At2g22880	−288
8	TCGGACCAA	III	rootsshoots1hr_2	At5g20230	−638
9	CTCTCTCAC	IV	roots0.5hr_8	At5g20230	−934
10	ATGTGATGC	V	shoots6hr12hr_2	At1g52410	−15
11	GCATCACCC	V	shoots6hr12hr_3	At2g43510	−873
12	CCAACTAA	VI	shoots12hr24hr_6	At2g43510	−355
12a	CAAACAAA	VI	shoots12hr24hr_6	At3g28220	−155/-924
13	TCTCCTCCAC	VII	shoots1hr_32	At5g45340	−49
14	CTTTCCCC	VII	rootsshoots0.5hr_36	At1g76650	−9
15	CCTCCTTCT	VII	rootsshoots0.5hr_21	At1g20510	−37/-745

Proveniences of selected motif sequences. Query refers to Figure 2. The genes and positions relative to transcription start refer to the genomic context of the cloned *cis*-elements.

**Table 2:** **Yeast One-Hybrid Screening with *cis*-sequences**

***Seq.***	***Cis*-sequence**		**Interacting transcription factors**	
**Nr**	**Group**	**Sequence**	**Main family**	**All families**
1	I	AACGTGGG	N.D.	bHLH (1), GATA (1), MADS (1), 3R-MYB (1), NAC (1)
2	I	GCACGTGGAG	bHLH (7/7)	bHLH (7)
3	II	GCTGCCGGAGA	NAC (2/2)	NAC (2)
4	II	GCCACGTCAGC	NAC (1/1)	NAC (1)
5	III	GGTTGTGGT	MYB (14/14)	MYB (14)
6	III	ACCAAACAT	MYB (4/4)	MYB (4)
7	III	CACCTAAC	MYB (16/16)	MYB (16)
8	III	TCGGACCAA	None	-
9	IV	CTCTCTCAC	bZIP (2/3)	bZIP (2), NAC (1)
10	V	ATGTGATGC	bHLH (4/4)	bHLH (4)
11	V	GCATCACCC	N.D.	G2-like (1), Zn finger (1)
12	VI	CCAACTAA	MYB (17/17)	MYB (17)
13	VII	TCTCCTCCAC	N.D.	bHLH (1), bZIP (1), NAC (1)
14	VII	CTTTCCCC	N.D.	C3HC4 (1), MYB (1), WRKY (1)
15	VII	CCTCCTTCT	None	-

N.B.: MYB refers to the R2R3-MYB class of MYB TF.

**Table 3:** Summary of the interactions identified and verified in yeast one-hybrid experiments

		**Studied *cis*-sequences**		**Identified transcription factors**		
**Motif group**		**Screened sequence**	**Mutated sequence**	**Gene ID**	**Family**	**Name**
I		AACGTGGG	AAC**C**TCGG	AT3G09370	3R-MYB	MYB3R3
I		AACGTGGG	AAC**C**TCGG	AT5G43650	bHLH	bHLH92
I		AACGTGGG	AAC**C**TCGG	AT4G32890	GATA	GATA9
I		AACGTGGG	AAC**C**TCGG	AT5G51870	MADS	AGL71
I		AACGTGGG	AAC**C**TCGG	AT3G12910	NAC	-
I		GCACGTGGAG	GCA**GC**TGGAG	AT1G59640	bHLH	bHLH031, ZCW32
I		GCACGTGGAG	GCA**GC**TGGAG	AT2G42300	bHLH	bHLH048
I		GCACGTGGAG	GCA**GC**TGGAG	AT2G18300	bHLH	bHLH064, HBI1
I		GCACGTGGAG	GCA**GC**TGGAG	AT5G61270	bHLH	bHLH072, PIF7
I		GCACGTGGAG	GCA**GC**TGGAG	AT1G10120	bHLH	bHLH074
I		GCACGTGGAG	GCA**GC**TGGAG	AT5G62610	bHLH	bHLH079
I		GCACGTGGAG	GCA**GC**TGGAG	AT1G51070	bHLH	bHLH115
II		GCTGCCGGAGA	GCTG**A**C**T**GAGA	AT2G43000	NAC	ANAC042, JUB1
II	**x**	GCTGCCGGAGA	GCTG**A**C**T**GAGA	AT3G12910	NAC	-
II		GCCACGTCAGC	GCCA**TA**TCAGC	AT3G12910	NAC	-
III		GGTTGTGGT	GGT**AC**TGGT	AT5G52260	R2R3-MYB	MYB019
III	**x**	GGTTGTGGT	GGT**AC**TGGT	AT1G74650	R2R3-MYB	MYB031, ATY13
III		GGTTGTGGT	GGT**AC**TGGT	AT3G48920	R2R3-MYB	MYB045
III		GGTTGTGGT	GGT**AC**TGGT	AT5G54230	R2R3-MYB	MYB049
III		GGTTGTGGT	GGT**AC**TGGT	AT1G57560	R2R3-MYB	MYB050
III		GGTTGTGGT	GGT**AC**TGGT	AT1G16490	R2R3-MYB	MYB058
III		GGTTGTGGT	GGT**AC**TGGT	AT1G08810	R2R3-MYB	MYB060
III		GGTTGTGGT	GGT**AC**TGGT	AT1G09540	R2R3-MYB	MYB061
III		GGTTGTGGT	GGT**AC**TGGT	AT1G56650	R2R3-MYB	MYB075, PAP1
III		GGTTGTGGT	GGT**AC**TGGT	AT5G26660	R2R3-MYB	MYB086
III		GGTTGTGGT	GGT**AC**TGGT	AT5G10280	R2R3-MYB	MYB092
III		GGTTGTGGT	GGT**AC**TGGT	AT3G47600	R2R3-MYB	MYB094
III		GGTTGTGGT	GGT**AC**TGGT	AT1G63910	R2R3-MYB	MYB103
III		GGTTGTGGT	GGT**AC**TGGT	AT3G02940	R2R3-MYB	MYB107
III		ACCAAACAT	AC**G**A**T**ACAT	AT2G47190	R2R3-MYB	MYB002
III		ACCAAACAT	AC**G**A**T**ACAT	AT3G24310	R2R3-MYB	MYB071, MYB305
III		ACCAAACAT	AC**G**A**T**ACAT	AT3G06490	R2R3-MYB	MYB108, BOS1
III		ACCAAACAT	AC**G**A**T**ACAT	AT1G48000	R2R3-MYB	MYB112
III		CACCTAAC	C**T**CCT**T**AC	AT2G47190	R2R3-MYB	MYB002
III		CACCTAAC	C**T**CCT**T**AC	AT5G52260	R2R3-MYB	MYB019
**III**	**x**	CACCTAAC	C**T**CCT**T**AC	AT1G74650	R2R3-MYB	MYB031, ATY13
III		CACCTAAC	C**T**CCT**T**AC	AT5G54230	R2R3-MYB	MYB049
III		CACCTAAC	C**T**CCT**T**AC	AT1G57560	R2R3-MYB	MYB050
III		CACCTAAC	C**T**CCT**T**AC	AT1G16490	R2R3-MYB	MYB058
III		CACCTAAC	C**T**CCT**T**AC	AT1G08810	R2R3-MYB	MYB060
III		CACCTAAC	C**T**CCT**T**AC	AT1G09540	R2R3-MYB	MYB061
III		CACCTAAC	C**T**CCT**T**AC	AT1G56160	R2R3-MYB	MYB072
III		CACCTAAC	C**T**CCT**T**AC	AT5G26660	R2R3-MYB	MYB086
III		CACCTAAC	C**T**CCT**T**AC	AT5G10280	R2R3-MYB	MYB092
III		CACCTAAC	C**T**CCT**T**AC	AT3G47600	R2R3-MYB	MYB094
III		CACCTAAC	C**T**CCT**T**AC	AT5G62320	R2R3-MYB	MYB099
III		CACCTAAC	C**T**CCT**T**AC	AT1G63910	R2R3-MYB	MYB103
III		CACCTAAC	C**T**CCT**T**AC	AT3G02940	R2R3-MYB	MYB107
III		CACCTAAC	C**T**CCT**T**AC	AT3G06490	R2R3-MYB	MYB108, BOS1
III		TCGGACCAA	TCGG**GT**CAA	None	-	-
IV		CTCTCTCAC	CTC**G**CTCAC	AT2G21230	bZIP	bZIP30
IV		CTCTCTCAC	CTC**G**CTCAC	AT1G06850	bZIP	bZIP52
IV	**x**	CTCTCTCAC	CTC**G**CTCAC	AT3G12910	NAC	-
V		ATGTGATGC	ATG**CA**ATGC	AT4G17880	bHLH	bHLH004, MYC4
V		ATGTGATGC	ATG**CA**ATGC	AT2G42300	bHLH	bHLH048
V		ATGTGATGC	ATG**CA**ATGC	AT2G18300	bHLH	bHLH064, HBI1
V		ATGTGATGC	ATG**CA**ATGC	AT5G61270	bHLH	bHLH072, PIF7
V		GCATCACCC	GCAT**AG**CCC	AT2G40970	G2-like	MYBC1
V		GCATCACCC	GCAT**AG**CCC	AT5G15840	Zn finger	CO
VI		CCAACTAA	CCA**GT**TAA	AT5G52260	R2R3-MYB	MYB019
VI		CCAACTAA	CCA**GT**TAA	AT1G74650	R2R3-MYB	MYB031, ATY13
VI		CCAACTAA	CCA**GT**TAA	AT5G06100	R2R3-MYB	MYB033
VI		CCAACTAA	CCA**GT**TAA	AT3G48920	R2R3-MYB	MYB045
VI		CCAACTAA	CCA**GT**TAA	AT5G54230	R2R3-MYB	MYB049
VI		CCAACTAA	CCA**GT**TAA	AT1G57560	R2R3-MYB	MYB050
VI		CCAACTAA	CCA**GT**TAA	AT1G16490	R2R3-MYB	MYB058
VI		CCAACTAA	CCA**GT**TAA	AT1G08810	R2R3-MYB	MYB060
VI		CCAACTAA	CCA**GT**TAA	AT1G09540	R2R3-MYB	MYB061
VI		CCAACTAA	CCA**GT**TAA	AT4G37260	R2R3-MYB	MYB073
VI		CCAACTAA	CCA**GT**TAA	AT2G26960	R2R3-MYB	MYB081
VI		CCAACTAA	CCA**GT**TAA	AT5G26660	R2R3-MYB	MYB086
VI		CCAACTAA	CCA**GT**TAA	AT5G10280	R2R3-MYB	MYB092
VI		CCAACTAA	CCA**GT**TAA	AT3G47600	R2R3-MYB	MYB094
VI		CCAACTAA	CCA**GT**TAA	AT4G21440	R2R3-MYB	MYB102, AtM4
VI		CCAACTAA	CCA**GT**TAA	AT1G63910	R2R3-MYB	MYB103
VI		CCAACTAA	CCA**GT**TAA	AT5G49330	R2R3-MYB	MYB111, PFG3
VII		TCTCCTCCAC	TCTC**GA**CCAC	AT5G61270	bHLH	bHLH072, PIF7
VII		TCTCCTCCAC	TCTC**GA**CCAC	AT2G21230	bZIP	bZIP30
VII		TCTCCTCCAC	TCTC**GA**CCAC	AT3G12910	NAC	-
VII		CTTTCCCC	CTT**GA**CCC	AT4G11680	C3HC4	-
VII		CTTTCCCC	CTT**GA**CCC	AT3G12720	MYB	MYB67
VII		CTTTCCCC	CTT**GA**CCC	AT5G43290	WRKY	WRKY49
VII		CCTCCTTCT	CCTC**AG**TCT	None	-	-

Bold characters: Mutated nucleotides; X: Interactions tested in transient assays using *Physcomitrella patens* protoplasts.

Within motif group I, two different sequences were assayed. For the first one, AACGTGGG, no specific class of TF was found to preferentially activate transcription in yeast. In this assay, five different TFs, belonging to five different families (*i.e.* bHLH, GATA, MADS, 3R-MYB and NAC), were identified (Table 3). Conversely, seven bHLH TFs (*i.e.* bHLH31, 48, 64, 72, 74, 79 and 115) were able to activate the transcription from the second sequence, GCACGTGGAG, revealing some transcriptional specificity (Table 3). Interestingly, both elements contain the ACGT core sequence, which once mutated (AAC**C**TCGG and GCA**GC**TGGA*G*) prevented the identified TFs to activate transcription in yeast, indicating its preponderant role in these interactions. The Y1H analysis of group V motifs, which belong to the cluster showing the highest level of sequence similarity with group I, led to the identification of four bHLH TFs. In fact, these bHLH factors were found to activate transcription in yeast from the ATGTGATGC sequence, but not from the GCATCACCC sequence, for which TFs from two other families (i.e. G2-like and Zn finger) were identified. The careful analysis of these group V motif sequences and their corresponding mutated versions ATG**CA**ATGC and GCAT**AG**CCC) exposed that when the ATGTGATGC sequence is concatenated, an E-box (CANNTG) is created, whereas this is not the case with the GCATCACCC sequence. The fact that sequences from group I and V display strong similarities with either the well described G ([GC] ACGT [GC]) or E boxes probably explains why bHLH TFs were found to be associated with these two motif groups. Group II is closely related, at the sequence level, to group I and V. However, unlike groups I and V, only two NAC TFs were found to interact with the two sequences tested (*i.e.* GCTGCCGGAGA and GCCACGTCAGC), with one (At3g12910) being identical for both sequences (Table 3).

From group III, four sequences were analysed, namely CACCTAAC, ACCACAACC (GGTTGTGGT), ACCAAACAT and TCGGACCAA. From these AC-rich sequences only R2R3-MYB TFs were identified as able to activate transcription in yeast (Table 3), with the exception of TCGGACCAA, for which no interacting TF was identified (Table 3).

The analysis of the CTCTCTCAC element from group IV concluded with the identification of three factors: one NAC and two bZIP (subgroup I) TFs ([38,39]; Figure 4E, Table 3).

Finally, three different sequences belonging to group VII were also analysed, namely TCTCCTCCAC, CTTTCCCC and CCTCCTTCT, from which no clear pattern of binding emerged. The screening of the TCTCCTCCAC sequence led to the identification of three different classes of TFs (bHLH, bZIP and NAC). Three additional factors belonging to three further classes (C3HC4, MYB and WRKY) where identified with the CTTTCCCC element. Finally, no binding TFs for the CCTCCTTCT element were retrieved from Regia in yeast (Table 3).

### Validation of transcription factor predictions with yeast-one-hybrid experimental data

**Figure 5:**
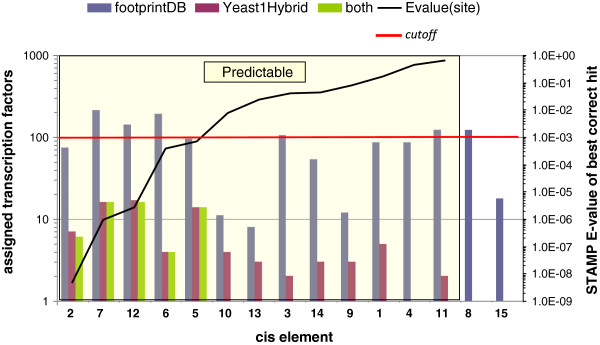
**Comparison between bioinformatically predicted and experimentally isolated TFs.***Cis*-elements were classified as ‘predictable’ (left) or as unpredictable (right) when the footprintDB repository contained at least one annotated TF homologous to the experimental Y1H-inferred binding proteins. Left vertical axis: columns report the number of TFs assigned to each tested *cis*-element by either footprintDB (blue) or the Y1H assay (purple), showing in green those assigned by both methods. Right vertical axis: line shows STAMP E-values for all 15 *cis*-elements aligned to the first hit validated by Y1H assays. Note that both vertical axes are in logarithmic scale.

In order to benchmark the accuracy of bioinformatically predicted candidate TFs, they were compared to those experimentally identified by Y1H screening. Key search parameters were tuned as explained in Methods and in Figure 3, and a trade-off between precision and sensitivity could be achieved. In summary, we were able to lower the BLASTP E-value threshold to 1E-10 without a significant sensitivity loss, but fixed the STAMP cutoff to 1E-3 to avoid a major sensitivity drop while ensuring reliable short *cis*-element alignments [30]. We also found that filtering out candidate TFs with poorly conserved interface residues, those directly contacting DNA nitrogen bases, was helpful in order to discard false predictions. A cutoff of 50% interface similarity was observed to be adequate, increasing specificity to 0.921, as shown in Additional file 4 [PredictionSummary]. Tested *cis*-elements were classified as predictable if the footprintDB repository contained at least one annotated TF with a significantly similar motif (STAMP E-value < 1E-3), which was homologous to the experimental Y1H-inferred factors. Thirteen of the 15 *cis*-elements tested in the Y1H experiment were accordingly called ‘predictable', but only 5 of them (2, 5, 6, 7, 12) yielded true positives among footprintDB results. The rest had significantly divergent DNA motifs annotated in the database, with E-values over the cutoff, and therefore could not be identified (see Figure 5). It is important to note that in all predictable cases the correct binding TFs were identified by homology to annotated plant regulatory proteins, including *A. thaliana*, *Petunia hybrida*, *Oryza sativa* or *Phaseolus vulgaris*. On the other hand, group II (sequence 4), III (sequence 8) and VII (sequence 15) elements could not be successfully matched as the IDEF2 and GCN4-like motifs (see Discussion) were not part of footprintDB when the predictions were made. Overall, after conducting the benchmark experiment using as input the 15 *cis*-sequences in Table 1, we conclude that we correctly predicted 56 out of 81 (69%) of the TFs isolated in the Y1H assays, finding on average 1 validated TF every 24 predictions. If only predictable sites are considered, these numbers improve to 56 out of 58 (97%) and 1 confirmed TF every 13 predictions, respectively. Finally, when the complete analysis was repeated with input DNA motifs (as produced by BEST) instead of individual *cis*-sequences, the fraction of successfully recovered TFs dropped to 39 out of 81 (48%), yielding 1 confirmed TF every 35 predictions. These results and the complete benchmark reports can be found in Additional file 4 [PredictionSummary].

### Functional analysis of TFs interacting with conserved *cis*-sequences

In this study, some factors were found to be able to activate transcription in yeast from various *cis*-sequences conserved in the promoters of genes that are simultaneously induced by drought. Amongst these, two were selected for more detailed analysis, NAC protein At3g12910 and MYB31 At1g74650. These selected TFs were able to induce transcription in Y1H experiments from three (group II, GCTGCCGGAGA and GCCACGTCAGC; group IV, CTCTCTCAC; and group VII, TCTCCTCCAC) and two (group III, CACCTAAC and ACCACAACC; and group VI, CCAACTAA) different motif groups, respectively (Table 3).

The ability of these two factors to interact with their DNA target *in vivo* was analysed in transient expression assays, using *Physcomitrella patens* protoplasts. In this experiment, the DNA binding domain (DBD) of the NAC protein and MYB31 was fused to the VP16 activation domain, resulting in the formation of two synthetic TFs. These chimeric proteins were then assayed against different conserved *cis*-sequences, namely CTCTCTCAC and GCTGCCGGAGA (TCTCCGGCAGC), and CACCTAAC and ACCACAACC, for the NAC and MYB31 DBD, respectively (Figure 4).

This analysis confirms the results obtained in Y1H experiments. Interestingly, the transcriptional activity supported by the NAC DBD was a lot stronger with the GCTGCCGGAGA sequence than with CTCTCTCAC, strengthening the idea that the GCTGCCGGAGA element is most probably a new NAC target sequence (Figure 4B, E). Conversely, similar activity was observed when MYB31 DBD was assayed against either, CACCTAAC or ACCACAACC, suggesting that MYB31 could activate the transcription from a wide variety of AC-rich *cis*-sequences (Figure 4C, D).

The *cis*-sequences and transcription factors identified in the present study may play a role in drought-responsive gene expression in *A. thaliana*. In the context of this assumption, several *cis*-sequences were used to generate synthetic promoters, which were tested in transgenic *A. thaliana* plants. However, no drought-responsive reporter gene expression has been observed in these transgenic lines so far (data not shown). It will be interesting to see if overexpression or knock-down mutations of the identified transcription factors will have any effect on drought-responsiveness of the plants.

## Discussion

The application of bioinformatic resources has led to the identification of large numbers of novel *cis*-regulatory sequences conserved in stress response genes [1-3]. Often the role of these elements and their binding transcription factors has remained unknown. The primary goal of the present work was the identification of TFs interacting with *cis*-sequences conserved in co-regulated genes. A total of 49 sequence motifs classified into 7 motif families were identified from microarray data on drought responsive gene expression. These motifs were bioinformatically predicted to interact with TFs belonging to specific TF families. These predictions were then tested experimentally using yeast one-hybrid screenings leading to the identification of novel TF-DNA interactions. Initially, the two bioinformatic approaches employed, preferably predicted TFs interacting with putative binding sites for motif group I that harbours 29 of a total of 49 motifs. Using the STAMP web server as well as footprintDB, these show significant similarities (low E-value) to G-box and ABRE elements. However, no consistency in TF predictions was obtained with these database-assisted analyses using the other motifs. This first set of results exposed the natural limitations of this approach, mainly associated with the data content of the underlying scanned databases. For example, predictions using the STAMP webserver depend on previously annotated data derived from databases such as TRANSFAC, JASPAR, PLACE, AGRIS, and AthaMap [23,28,40-42]. The content of these repositories is primarily derived from functionally known regulatory sequences, which are expected to be only a tiny fraction of all *cis*-elements. Similarly, footprintDB (http://floresta.eead.csic.es/footprintdb) contains TFs and their experimentally-associated DNA motifs from these and other databases. A main difference to the STAMP webserver is that footprintDB motifs are associated to their cognate binding TFs, and their DNA-binding interfaces are annotated using 3D-footprint structural data [29]. However, STAMP as well as footprintDB detect and predict TF-DNA interactions based on known data. Therefore, TF-DNA interactions not readily predicted with STAMP and footprintDB may represent novel or to date unknown TF-DNA interactions. This was substantiated in the present study by using sequences enriched in promoters of drought-responsive genes, but not readily associated with interacting TFs for experimental analysis using Y1H-screenings. Towards these ends two different sequences from motif group I were assayed. For the first one, AACGTGGG, no specific class of TF was found to preferentially induce transcription in yeast. Conversely, seven bHLH TFs were able to activate the transcription from the second sequence, GCACGTGGAG. Both elements contain the ACGT core sequence, which once mutated (AAC**C**TCGG and GCA**GC**TGGAG) prevented transcription to be activated. These observations suggest that sequences AACGTGGG and GCACGTGGAG are *bona fide* binding sites for bHLH31, 48, 64, 72, 74, 79 and 115. Motif group V showed the highest levels of sequence similarity with group I and drove the identification of four bHLH TFs. In fact, the bHLH TFs were found to prompt transcription in yeast from *cis*-element ATGTGATGC, but not from GCATCACCC. When sequence ATGTGATGC is concatenated, an E-box (CANNTG) is created (i.e. ATGTGATGCATGTGATGC), whereas this is not the case with GCATCACCC. The fact that sequences from group I and V contain or display strong similarities with either the well described G-box ([GC] ACGT [GC]) or E-box probably explains why bHLH TFs were found to be associated with these two motif groups. In fact, numerous studies have shown that bHLH TFs regulate the activity of their target gene promoters through these types of *cis*-regulatory sequences [43,44]. However, no bZIP TFs were identified as interacting with the motif that contains the ACGT core, suggesting that the interactions with the bHLH TFs are quite specific and that the full-length sequence of the motifs may have a role in this specificity [38]. Group II is closely related, at the sequence level, to group I and V. However, unlike group I and V, only two NAC TFs were found to interact with the two sequences tested (GCTGCCGGAGA and GCCACGTCAGC), with one (At3g12910) being identical for both sequences (Table 3). A wide variety of NAC binding sites have been identified so far in various plant species, revealing the large diversity of the sequences that can be recognised by this group of TFs [45-47]. The GCCACGTCAGC sequence displays similarities with the core sequence CA [AC] G [TC] [TCA] [TCA] that is recognised by the rice (*Oryza sativa*) and barley (*Hordeum vulgare* L.) IDEF2 NAC proteins, suggesting that the identified NAC could activate the transcription in yeast from it [47]. This assertion was supported by the lack of transcriptional activity associated with the mutagenesis of this element (GCCA**TA**TCAGC). Interestingly, the GCTGCCGGAGA sequence appears to be quite divergent when compared to the other described NAC binding sites even though both NAC TFs were able to activate the transcription in yeast from it, suggesting that the GCTGCCGGAGA DNA sequence could be a new *cis*-regulatory element that could be targeted by NAC proteins (Figure 4B).

From group III, the AC-rich sequences CACCTAAC, ACCACAACC (GGTTGTGGT), ACCAAACAT and TCGGACCAA identified only R2R3-MYB TFs to be able to activate transcription in yeast (Table 3). This result is in agreement with previous studies that have demonstrated that numerous R2R3-MYB proteins from different plant species are able to bind to and induce transcription from AC-rich sequences [48-50]. One of the studied sequences (CACCTAAC) contains the well-described AC-I *cis*-regulatory element (ACCTAAC) to which the *A. thaliana* MYB61 TF (R2R3-MYB subgroup 13) was shown to bind to *in vitro* and activate transcription from it in yeast [50]. This result was confirmed in our screen (Figure 4C). Similarly, we found that two other R2R3-MYB subgroup 13 members (composed of four TF genes), namely MYB50 and MYB86, were also able to activate transcription in yeast from the same element [51]. The overlap between the R2R3-MYB proteins that interact with the CACCTAAC (16 interacting R2R3-MYB) and the ACCACAACC (14 interacting R2R3-MYB) sequences was quite high, as 12 out of the 18 interacting R2R3-MYBs (67%) were common to both elements (Table 3, Figure 4C, D). Interestingly, only four R2R3-MYBs prompted transcription in yeast from the ACCAAACAT element, from which three belong to the same R2R3-MYB subgroup, namely subgroup 20 (composed of six members) [51]. Group VI, such as Goup III, is composed of AC-rich motifs. From this motif group only one putative *cis*-regulatory sequence was assayed, namely CCAACTAA, from which only R2R3-MYB TFs (17 genes) were also identified (Table 3). Eleven (64,7%) and 12 (70,6%) of the identified R2R3-MYBs were identical with the R2R3-MYBs found to interact with the CACCTAAC and ACCACAACC sequences, respectively. When concatenated, this motif partially contains the R2R3-MYB targeted AC-II element (ACCAACC) from which the identified TFs are likely to induce transcription in yeast, based on the absence of activation with the mutated version (**GTT**AAC) of the element [48].

Surprisingly, as no ACGT core sequence (A-box, C-box or G-box) is present in this motif, the analysis of the CTCTCTCAC element from group IV leads to the identification of two bZIP (subgroup I) factors ([38,39]; Figure 4E, Table 3). Nevertheless, a detailed analysis of this sequence showed that a bZIP-like DNA target (GGTGAGAGAG) similar to two GCN4-like (GGTGAG and TGTGTGACA) motifs found in the promoter of the wheat storage protein genes was present, suggesting that this sequence could be mainly recognised by this class of bZIP factors [52].

While these Y1H results on their own already provide valuable biological insight, they also served in this work as a reference dataset. Indeed they were also used to benchmark the predictive value of the footprintDB approach for recognizing putative binding TFs with different parameter settings. Two STAMP E-value cutoffs were tested. While a stringent 1E-5 threshold yielded the most specific predictions, it was at the cost of a significantly reduced sensitivity (52%). On the contrary, a relaxed 1E-3 cut-off increased the sensitivity (78%) by compromising specificity. However, this loss of specificity could be corrected by lowering the BLASTP E-value cut-off to 1E-10. In addition, we observed a further gain in specificity by applying an interface similarity filter for candidate TFs, requiring them to have at least 50% similar interface residues to be considered. Altogether, these settings yield a 69% sensitivity and a 92% specificity. Another important observation regarding the performance was that individual input *cis*-elements worked much better than sequence motifs, which presumably increase the chance of obtaining significant but irrelevant alignments of DNA sequences, which are the cornerstone of this approach.

In addition to the Y1H screenings, the *in vivo* interaction of two TFs with their DNA target was analysed in transient expression assays using *Physcomitrella patens* protoplasts. In this experiment, two synthetic TFs were generated by fusing the DNA binding domain (DBD) of the NAC protein (At3g12910) and MYB31 (At1g74650) to the VP16 activation domain. These chimeric proteins were then assayed against the conserved *cis*-sequences, CTCTCTCAC and GCTGCCGGAGA, and CACCTAAC and ACCACAACC, for the NAC and MYB31 DBD, respectively (Figure 4). Interestingly, the transcriptional activity supported by the NAC DBD was a lot stronger with the GCTGCCGGAGA sequence than with CTCTCTCAC one, strengthening the idea that the GCTGCCGGAGA element is most probably a new NAC target sequence (Figure 4B, E). To date the NAC protein encoded by the *Arabidopsis* gene At3g12910 had not been investigated experimentally. Comparing the proposed novel NAC binding site GCTGCCGGAGA with known *Arabidopsis* NAC binding sites reveals a large variability of binding sites recognised by NAC proteins. For example, the NAM (At1g52880) and NAP (At1g69490) proteins bind to the *cis*-sequences AAGGGATGA and CACGTAAGT, respectively [53,54]. The high variability of NAC binding sites is also illustrated by the A-rich binding site of ATAF2 [55]. NAC transcription factors are involved in a wide array of abiotic stress responses [56]. Interestingly, the NAC transcription factors ANAC019 (At1g52890), ANAC055 (At3g15500), and ANAC072 (At4g27410) bind to the sequence ANNNNNTCNNNNNNNACACGCATGT*,* a drought responsive *cis*-sequence [45]. Although this sequence is part of a drought responsive promoter (*ERD1*), overexpression of ANAC019, ANAC055, and ANAC072 in transgenic plants did not up-regulate *ERD1* expression indicating that other interacting factors may be necessary for the induction of the *ERD1* gene [45]. In this context it is interesting to note that none of the *cis*-sequences investigated here, although enriched in drought responsive genes, individually confer drought responsive gene expression (data not shown). Among these sequences was also the novel NAC binding site GCTGCCGGAGA. Similarly it may be suggested that the investigated *cis*-sequences are each part of a combinatorial element that requires a second binding site for an interacting transcription factor in close vicinity for its functionality [57].

When the MYB31 DBD was assayed against CACCTAAC and ACCACAACC in *Physcomitrella patens* protoplasts using a synthetic TF, similar activity was observed with both *cis*-sequences, suggesting that MYB31 could induce transcription from a wide variety of AC-rich *cis*-sequences (Figure 4C, D). MYB31 belongs to subgroup 1 of R2R3MYB TFs [51]. The gene is known to be up-regulated by chitin but no further functional analysis has been carried out to date [58]. There are 126 R2R3-MYB TFs in the genome of *Arabidopsis*[51] and random binding site selection experiments with MYB15, 61, 77, and 84 revealed that these factors always bind to *cis*-sequences with one or two conserved ACC and/or AAC core sequences [59,60]. Consequently, the adjacent nucleotides can vary significantly and *Arabidopsis* MYB factors have a relatively degenerate binding site recognition. Consistent with known MYB binding sites, the synthetic TF containing the MYB31 DBD activates gene expression from two synthetic promoters harbouring the ACC and AAC motif (CACCTAAC and ACCACAACC). Although the MYB31 binding site CACCTAAC does not confer drought responsive reporter gene expression in transgenic *Arabidopsis* (data not shown), this motif has been found in *Arabidopsis* in the promoter of three MYB61 target genes that are involved in vasculature development, one of the main components of the transpiration stream, which indirectly may participate in plant adaptation to drought stress [50]. Such observations reinforce the postulate that a combination of regulatory elements is required to integrate the environmental signals on a specific gene promoter [57].

## Conclusions

The work presented demonstrates the successful integration of several bioinformatic resources to predict and validate TFs interacting with conserved sequence motifs in co-regulated genes. Predictions were confirmed by using a yeast-one-hybrid approach to identify interacting TFs belonging to the predicted TF families. TF-DNA interactions were further experimentally validated in yeast and with a *Physcomitrella patens* transient expression system, leading to the discovery of several novel TF-DNA interactions. Our work establishes a novel approach to identify TFs interacting with conserved *cis*-sequences. This approach may facilitate the experimental identification of TFs because a candidate TF-family can be predicted bioinformatically using the footprintDB database. Although the limitation of this approach is the content of the databases used, footprintDB is particularly valuable because it contains data for TF-DNA interactions from many different species. Therefore, this database may help to identify a DNA binding protein domain for any submitted sequence. Afterwards one may check plant TF databases for the predicted DNA binding domain to identify novel sequence-specific TF-DNA interactions.

## Methods

### Bioinformatic analysis to identify conserved *cis*-sequences in co-regulated genes

*In silico* identification and bioinformatic analysis of sequence motifs overrepresented in drought stress-induced *A. thaliana* genes was based on microarray expression data from the AtGenExpress global stress expression data set [61]. To identify genes up-regulated by drought, Affymetrix ATH1 microarray raw expression data were downloaded from NASCArrays (NASCARRAYS-141) [62], normalised using MAS5 algorithm [63], and scaled to a TGT of 100. An untreated control data set (NASCARRAYS-137) was also downloaded and identically processed. Array elements were assigned to genes according to an assignment table based on TAIR release 8 annotations [64]. Expression data were imported into the PathoPlant database [20]. The annotation procedure of cDNA microarray data and Affymetrix ATH1 data has been described earlier [3,21]. All data as well as links to the microarray source of the expression set can be found on the PathoPlant site at http://www.pathoplant.de/documentation.php.

Expression data was used to identify genes up-regulated upon drought stress. Genes showing at least a 2-fold induction compared to untreated control were defined as up-regulated. Using an SQL server query tool, 32 drought stress combinations at different time points and in different tissues were queried. PathoPlant’s ‘Microarray expression’ online tool displays a similar functionality to the query tool described above and can be used to determine sets of genes co-regulated upon drought.

For promoter analyses of drought-induced gene sets, sequences 1,000 nt upstream of the transcription start site, if known as for the majority of genes, otherwise 1,000 nt upstream of the ATG start codon were extracted using TAIR release 8 sequence annotation and converted into FASTA format. To identify overrepresented motif sequences within these promoters, the BEST software package [19], locally installed on a Linux SuSE9.2 system was used [3]. The package combines 4 different motif-finding programs (MEME, AlignACE, CONSENSUS, BioProspector) and an optimization step. BEST was run with default parameters and predefined motif lengths of 5 to 10 nucleotides. The application of these parameters had previously shown to yield optimal results with promoter sequences from *A. thaliana*[3]. Overrepresented sequence motifs identified by BEST were further used if detected by at least 2 out of the 4 motif finding programs.

Such a BEST analysis with 33 gene sets yields a high number of enriched sequence motifs. In order to pre-validate these bioinformatically, a so-called *in silico* expression analysis was performed with all motif sequences (http://www.pathoplant.de/expression_analysis.php). This analysis is based on PathoPlant’s microarray expression database and correlates motif sequence occurrences with stress-specific gene expression data resulting in an evaluation of the sequence to identify the stress this sequence is most likely to be responsive for [31]. Strict correlation criteria were applied by only considering motif sequences that displayed at least two stresses associated with drought among the first 3 highest ranked stresses.

Identified sequence motifs in which at least one sequence was found to be enriched in promoters of drought stress responsive genes were submitted to the STAMP web server applying the recommended default parameters [22,40]. STAMP classified all motifs based on matrix alignment to a similarity tree given in Newick tree format that was displayed using MEGA [32]. Groups containing similar motifs were generally defined by clustering single motifs on branches with lengths <0.05. In one exceptional case a motif (shoots3hr_1) was grouped into motif group II based on its conserved core sequence GCC that is characteristic for AP2/EREBP binding proteins. This core sequence is also conserved in the other two motifs of motif group II. STAMP was also employed for the identification of motif similarities by comparison with known *cis*-elements from plant databases AthaMap, AGRIS and PLACE [23-25].

### Bioinformatic prediction of candidate transcription factors for selected *cis*-elements

Sequence motifs obtained for drought stress related genes were used as queries for footprintDB (http://floresta.eead.csic.es/footprintdb). This repository currently contains over 5,000 unique TFs and their DNA binding preferences annotated as position-specific scoring matrices (PSSMs) that capture the occurrence of nucleotides in different positions of the DNA binding site [65]. When building footprintDB for this analysis, data were extracted from the literature and other repositories such as TRANSFAC, JASPAR, 3D-footprint, UniPROBE, AthaMap, DBTBS, RegulonDB [12,29,40,41,66-68], and several papers reporting human, murine, and Drosophila motifs [69,70].

The footprintDB engine searches for similar motifs using STAMP [22]. The main difference to the STAMP webserver is that footprintDB motifs are associated to their cognate binding TFs, and their DNA-binding interfaces are annotated using 3D-footprint structural data [29]. Interface residues are defined for being located within 4.5 Angstroms with respect to at least one DNA nitrogen base in homologous structural complexes deposited at the Protein Data Bank. By taking advantage of these data, footprintDB is able to predict *A. thaliana* homologous TFs that are candidates to bind the input *cis*-sequences, calculating interface similarity with a custom scoring matrix motifs [30]. The search parameters were: 1) STAMP E-value: 1E-3, 2) BLASTP E-value: 1E-10, 3) *Arabidopsis* protein source: TAIR9, containing 33,410 protein sequences [64], 4) Interface similarity cut-off: 50%.

### Yeast one-hybrid (Y1H) experiments

All the primers used in this section were purchased from Sigma-Aldrich (Lyon, France) and are listed in Additional file 6.

The putative *cis*-regulatory sequences identified in this study were synthesised as hexamers, cloned (*EcoR*I and *Xba*I) into the pHISi vector (Clontech, Saint-Germain-en-Laye, France) and stably transformed into yeast (*Saccharomyces cerevisiae*, EGY48α-type mating strain) at the *URA3* locus. Fifteen different *cis*-sequences, from seven motif subgroups, that were not inducing self-activation in yeast and that were preferentially not already described in the literature as associated with drought stress response, were selected for experiments. For this purpose, a yeast (YM4271, a-type mating strain) normalised cDNA library of *A. thaliana* TFs cloned into *pDEST22*™ (Invitrogen, Saint Aubin, France) was used (REGIA library [71]).

Following the mating of both yeast strains, diploid colonies growing on a medium lacking the histidine amino acid were considered as positive clones expressing the candidate TFs interacting with the studied sequences. Then, a verification step was carried out, which consisted in testing the candidate TF’s activity from both, their target sequence, and their corresponding mutated version. When yeast growth was observed from the unmodified sequence and not from the mutated version, the corresponding TF was considered as positive interactor. In order to increase the stringency of the screens, various concentrations of 3-aminotriazol (3-AT) were used in all the Y1H experiments (from 15 to 60 mM). The details of the yeast transformation, mating and selection are reported elsewhere [72].

Finally, for each of the confirmed interactions, the expressed cDNA was amplified from the diploid colonies by PCR and the resulting amplicons were subsequently sequenced in order to confirm the identity of the identified TFs. In order to facilitate the PCR reaction, the yeast cell wall was hydrolysed by a lyticase (Sigma-Aldrich, Lyon, France) treatment. Briefly, yeast cells were suspended in 15 μl lyticase solution (2 mg/ml in 0.1 M sodium phosphate buffer, pH 7.5), incubated 30 min at 37°C and then 10 min at 95°C (heat inactivation), and finally diluted by adding 85 μl of sterile water. Five μl lysate was then used per 50 μl PCR reaction.

### Bioinformatic validation of transcription factor predictions with yeast-one-hybrid experimental data

Y1H experimental results allowed us to assess the performance of footprintDB transcription factor predictions and to tune the search parameters to increase its effectiveness. The set of 15 *cis*-sequences used for Y1H screenings was analysed in order to predict putative *A. thaliana* binding TFs. Different strategies and parameter combinations were tested and their impact on the results was evaluated, since we observed that the systematic comparison of short DNA sequences, such as *cis*-elements, was troublesome [73]. Two *A. thaliana* protein sequence sources were tested: the list of 995 TFs cloned in the Y1H library (from REGIA) and the TAIR9 longest transcript proteome [64]. The resulting optimum strategy was employed in order to predict putative binding TFs. Parameter setting evaluation and the complete predictions are reported in Additional file 4. This file contains several sheets: 1) the data supporting Figure 5; 2) a table with performance measurements of footprintDB with different parameter settings [PredictionSummary]; 3) the complete set of predictions for input motifs [Prediction (motifs)]; 4) the complete set of predictions for input individual DNA sites [Prediction (sites)]; 5) A file with all *A. thaliana* homologous TFs and their annotations [A.th.homologues]; and 6) a summary of significantly similar motifs found within footprintDB for each input DNA motif [MotifSimilarity].

### *Physcomitrella patens* transfection assays

All the primers used in this section were purchased from Sigma-Aldrich (Lyon, France) and are listed in Additional file 6.

Four sequences belonging to three different motif groups were assayed against DNA binding domains from two TFs (*NAC*, At3g12910 and *MYB31*, At1g74650) fused to the VP16 activation domain in *P. patens* protoplast transient expression assays. These sequences were synthesised as hexamers fused to the 35S cauliflower mosaic virus minimal promoter and recombined into the *pBS TPp-B* vector [74]. The NAC and MYB31 VP16-fusions were carried out by PCR using the high-fidelity Phusion DNA polymerase with the HF buffer (Thermo Fisher Scientific, Villebon sur Yvette, France) prior to recombination into the *pBS TPp-A* vector [74]. Gateway® recombination, protoplast transformation and quantitative analysis of reporter gene expression are described in detail elsewhere [74].

## Competing interests

The authors declare that they have no competing interests.

## Authors’ contributions

CD, ZK, GH, WX, DG, FS, and CB performed the experiments. AS, BCM, and LB performed the bioinformatic analysis. CD, ZK, GH, AS, BCM, LB, and RH wrote the paper. CD, LL, BW, BCM, LB, and RH designed the work. All authors read and approved the final manuscript.

## Supplementary Material

Additional file 1 Parameters for PathoPlant database queries and number of induced genes obtained in each of the 32 queries. Click here for file

Additional file 2 Sequences and their alignments generating the 49 sequence motifs. Click here for file

Additional file 3 **Similarities of motifs to known *cis*-regulatory sequences in the AthaMap, AGRIS, and PLACE databases.** Click here for file

Additional file 4 Results of the footprintDB analyses. Click here for file

Additional file 5 Results of the Y1H screen. Click here for file

Additional file 6 **Primers used for the Y1H screen and the *Physcomitrella patens *** **transfection assays.** Click here for file
